# A detailed systematic anatomical study of monocephalic conjoined symmetric twin lambs

**Published:** 2014-12-13

**Authors:** Y. Kamali, Z. Khaksar, A.R. Sharaki, B. Rasouli, M. Kajbafi

**Affiliations:** 1 *Department of Basic Sciences, School of Veterinary Medicine, Shiraz, Iran*; 2 *Department of Clinical Sciences, School of Veterinary Medicine, Shiraz, Iran*

**Keywords:** Monocephalus, Sheep, Tetrabrachius, Tetrascelus, Thoracopagus

## Abstract

A case of conjoined twins with monocephalus, thoracopagus, partial abdominopagus, tetrabrachius and tetrascelus in lambs complicated with several defects of skeletal, cardiovascular, gastrointestinal, and urogenital systems is reported. The twins were dead and delivered by cesarean section. This case report highlights the detailed anatomical study of defects in different systems due to an abnormal birth defect.

## Introduction

Under normal circumstances, both members of a twin pair are completely separated. Sometimes the separation is not complete and the twins remain conjoined to various degrees and at almost any site of their bodies (Hyttel *et al.*, 2010).

Conjoined twinning has been reported in most domestic animal species. It occurs rarely in horses, occasionally in dogs and cats (Saperstein, 1981; Fisher *et al.*, 1986), and more commonly in cattle than in other ruminants (Leipold *et al.*, 1972; Pal and Verma, 1981; Ramadan, 1996). In sheep, the failure of separation most commonly involves the cranial region, whereas in cattle, the defect is most typically confined to the caudal region (Dennis, 1975).

### Case details

**Fig. 1:**
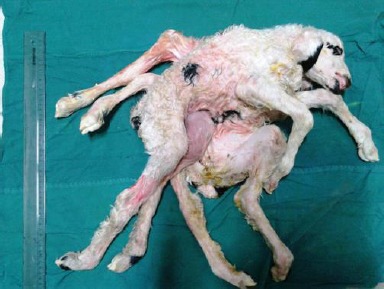
External view of twin lambs showing a single head and duplication of the forelimbs, hind limbs, and tail.

A pregnant ewe was referred to the Veterinary Teaching Hospital of Shiraz University, Iran, for dystocia. The twins were the first dam offsprings. They were conjoined together and were delivered by cesarean section. Both of them were male and dead before the parturition. Twin lambs had joined chests with a single head (Fig. 1).

**Fig. 2:**
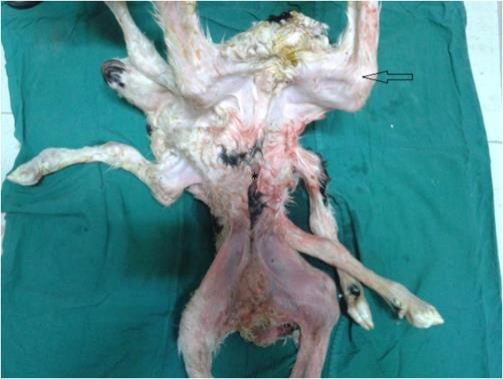
Abdominal region of twin lambs showing only one umbilicus (*) tend to the left twin that indicated by arrow.

**Fig. 3:**
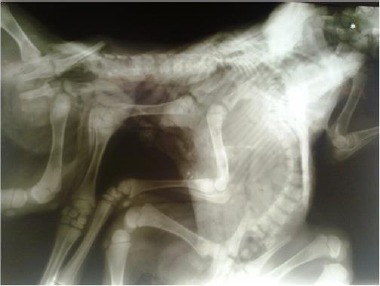
Plain radiography showing bifurcation of vertebral columns at the level of C3 (*). Also 2 sets of ribs and 2 sterna but only a common thoracic cavity.

Interestingly, the cranial parts of the abdomens were fused, whereas the caudal parts were separated (partial abdominopagus). As a result, only one umbilicus and one mixed umbilical cord with a tendency toward the left one was formed (Fig. 2). There was no history of other defects within the flock. To evaluate the skeletal structure, radiographic examination was performed. Plain radiography revealed bifurcation of vertebral columns at the level of C3 (Fig. 3).

Consequently a detailed dissection was done as an anatomical survey. The dam and lambs were not examined for congenital anomalies caused by viruses.

**Fig. 4:**
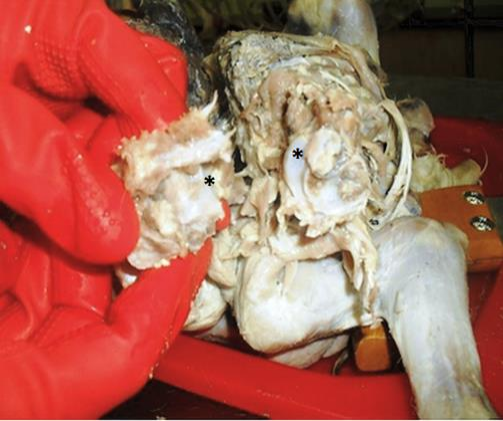
Cranial view of the separated axes showing articular surfaces (*) between them medially.

The conjoined twins had a single normal head with normal structures; the occipital bone also had a single foramen magnum. In the cervical region, there was one atlas with two vertebral foramens, and paired axes articulated with each other medially (Fig. 4).

**Fig. 5:**
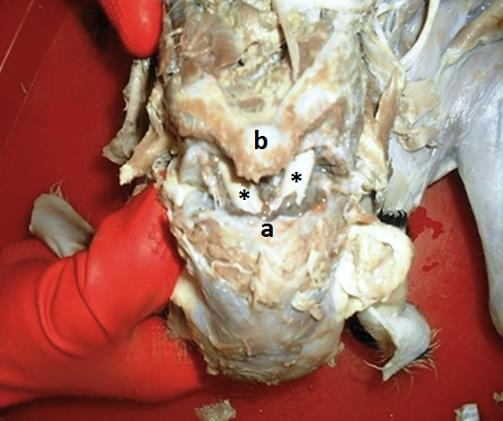
Dorsal aspect showing bifurcation of the spinal cord at the site of atlanto-occipital joint. (*): Spinal cord; (a): Occipital bone; (b): Atlas.

The rest of the vertebral column was bifurcated at the C3 and extended caudally to include 2 distinct tails, whereas bifurcation of the spinal cord was just caudal to the foramen magnum (Fig. 5).

There were 8 limbs: 4 brachial (Tetrabrachius) and 4 pelvic (Tetrascelus). One forelimb of each twin was situated on the normal site, but the other one located along the vertebral column and attached to its counterpart, belonging to the other twin by a common brachiocephalic muscle.

There were also 2 sets of ribs and 2 sterna but only a single thoracic cavity was formed, which widened craniocaudally.

The cavity was bound ventrally by a seemingly normal sternum, laterally by the 13 pairs of ribs, caudally by a broad diaphragm, separating the thoracic cavity from the abdomen, and dorsally by vertebral columns, smaller ribs, and the tip of the second sternum. The latter which passed caudad and laterad, was located between the diverging vertebral columns, deviated to the left side, and articulated with some additional ribs from the other twin. The xiphoid cartilage was attached to the hypoplastic diaphragm.

**Fig. 6:**
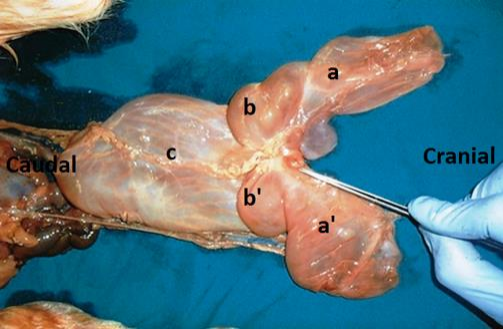
Dorsal view of the double proventriculi and a single abomasum showing (a): Right rumen; (a’): Left rumen; (b): Right reticulum; (b’): Left reticulum; (c): Single abomasum. The point of the scalpel indicates the termination of the esophagus.

There was one esophagus, 1 heart, 1 trachea, 2 lungs and 2 vagus nerves in the thoracic cavity. The esophagus after passing of a seemingly normal pathway was terminated at the cardiac orifice, situated at the fusion site of the double proventriculi, a little to the left side of the median plane (Fig. 6).

The lungs were smaller than usual, but their lobations were normal. Regarding the heart structure, except for a slightly dilated aortic orifice, no other defects were observed. Also, the cardiac septation was fully and normally developed. The left and right ascending aortae were fused together from their origins and formed a common stout trunk and continued cranially as a single brachiocephalic trunk and the right subclavian artery.

**Fig. 7:**
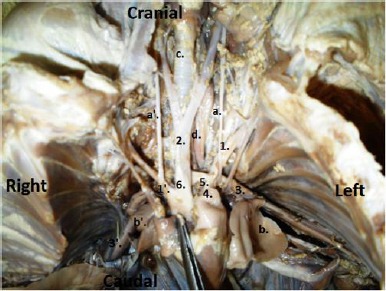
Ventral view of the thorax and neck showing (1): Left subclavian a.; (1’): Right subclavian a.; (2): Bicarotid trunk; (3): Left descending aorta; (3’): Right descending aorta; (4): Pulmonaray trunk; (5): Ductus arteriosus; (6): Brachiocephalic trunk; (a): Left vagus; (a’): Right vagus; (b): Left lung; (b’): Right lung; (c): Trachea; (d): Esophagus.

The left subclavian artery was branched separately from the convexity of the left aortic arch. Interestingly, two subclavian arteries, the left and right, were arised caudally from the origins of the corresponding descending aortae. Only one pulmonary trunk was observed in the left side and that was connected to the left aortic arch by a single ductus arteriosus (Fig. 7).

**Fig. 8:**
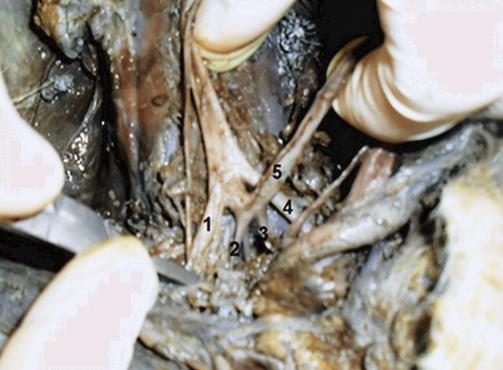
Ventral view of the right twin near the pelvic inlet showing (1): Right external iliac a.; (2): Right internal iliac a.; (3): Left internal iliac a.; (4): Left external iliac a.; (5): Umbilical a.

**Fig. 9:**
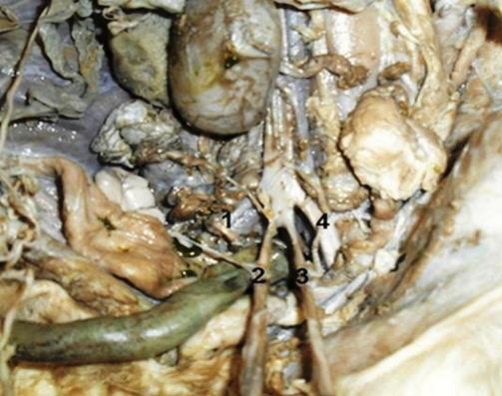
Ventral view of the left twin near the pelvic inlet showing (1): Right external iliac a.; (2): Right umbilical a.; (3): Left umbilical a.; (4): Left external iliac a.

In the left twin there was one pair of the umbilical arteries, but in the right twin only one single umbilical artery was observed (Fig. 8 and 9).

In the abdominal cavity, a complete duplication of spleen and liver was found which the right liver was larger than the left one.

Double reticuli and rumens externally were fused together in the midline, the omasi were under grown and a complete fusion of the two abomasi had resulted in formation of a single abomasum (Fig. 6).

**Fig. 10:**
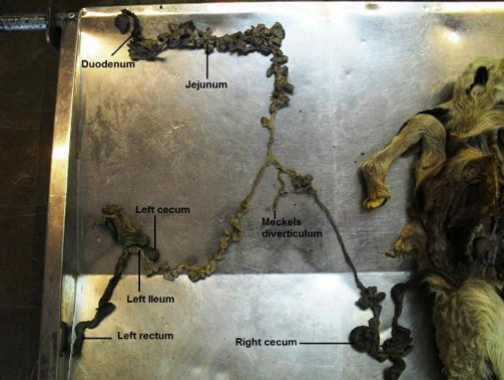
A complete duplication of intestines from midway of jejenum toward anus.

Two spleens were separately attached on either side of the fused rumens. The intestines from the pyloric portion to midway of the jejunum were single, but afterwards a complete duplication of the ileum, colon, rectum and anus was seen (Fig. 10).

**Fig. 11:**
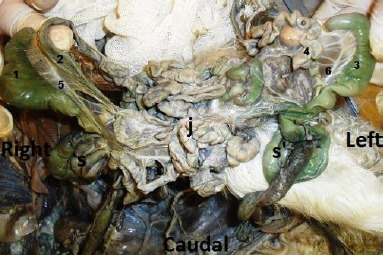
Abdominal cavity showing (1): Right cecum; (2): Right ileum; (3): Left cecum; (4): Left ileum; (5): Right ileocecal fold; (6): Left ileocecal fold; (s): Right spiral loop; (s’): Left spiral loop; (j): Jejenum.

**Fig. 12:**
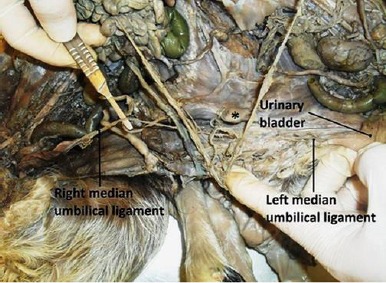
Ventral view of the caudal part of the twin abdomens. The right median umbilical ligament has extended to the other side, and the left one is in normal site. The star shows the xiphoid cartilage of the second sternum.

The jejunum had numerous close coils around the border of the common mesentery and was irregularly positioned between the two colons in the center of the common abdomen (Fig. 11). No atresia ani was observed. Further dissection also revealed 2 pairs of kidneys and testes; one testis from each twin was retained in the abdomen. The median umbilical ligament of the right twin was extended from the corresponding apex of urinary bladder to the umbilicus of the left twin due to the lack of right twin umbilicus (Fig. 12).

## Discussion

The frequency of congenital malformations varies with species, breed, geographical locations and due to many other factors. Various studies indicate that approximately 1.5% to 6% of all live-born domestic mammals show at least one recognizable congenital malformation (Hyttel *et al.*, 2010).

Such malformations are comparatively infrequent in cats but occur to an upper limit of 3–4% in sheep, cattle and horses and up to 6% in newborn dogs and pigs (Hyttel *et al.*, 2010). The genesis of congenital malformations is best regarded as an interaction between the genetic constitution of an embryo and the environment in which it develops.

In contrast to human medicine, in the veterinary literatures, sufficient evidence regarding congenital malformations is not available. Multiple births most frequently result from fertilization of separately ovulated female gametes. However, partial splitting of the primitive node and streak may result in the formation of conjoined (Siamese) twins.

Conjoined or fused symmetric twins are classified according to the nature and degree of union as thoracopagus (pagos, fastened); pygopagus; craniopagus and ischiopagus (Noden and De Lahunta, 1985). When these twins have a single head, it is called monocephalus. In this case, the failure of separation involved most structures cranial to C3, leading to the appearance of duplication of both the axial and ab-axial portions of the skeleton and associated organs. This particular combination has only been reported in a dog (Nottidge *et al.*, 2007).

Our case is similar to the conjoined twin lambs reported in 2010 (Spiers *et al.*, 2010), but there are some differences and new additional findings. The causality of birth defects is not necessarily genetic in origin and various aetiological categories can be recognized: Chromosomal anomalies, Polygenic disorders, Single gene mutations, Environmental/teratogenic factors, Multifactorial aetiology and Unknown aetiology.

The embryo does not occupy a completely protected and privileged environment and, in some respects, is as open to effects from its environment as the neonate or adult. Also, teratogenic materials in one species do not necessarily cause malformation in other species.

The present case has been under intensive care during the pregnancy based on the owner’s statements. Hence, the anomaly does not seem to be caused by grazing freely.

The reason for this anomaly in this sheep could not be ascertained, as no further tests or assays were carried out.
